# Portable and cost-effective genetic detection and characterization of *Plasmodium falciparum hrp2* using the MinION sequencer

**DOI:** 10.1038/s41598-022-26935-z

**Published:** 2023-02-18

**Authors:** Susanna Sabin, Sophie Jones, Dhruviben Patel, Gireesh Subramaniam, Julia Kelley, Michael Aidoo, Eldin Talundzic

**Affiliations:** 1 Malaria Branch, Division of Parasitic Diseases and Malaria, Center for Global Health, Centers for Disease Control and Prevention, Atlanta, GA USA; 2 Oak Ridge Institute for Science and Education, Oak Ridge, TN USA; 3 Williams Consulting, Catonsville, MD USA

**Keywords:** Microbial genetics, Parasite genetics

## Abstract

The prevalence of *Plasmodium falciparum hrp2* (*pfhrp2*)-deleted parasites threatens the efficacy of the most used and sensitive malaria rapid diagnostic tests and highlights the need for continued surveillance for this gene deletion. While PCR methods are adequate for determining *pfhrp2* presence or absence, they offer a limited view of its genetic diversity. Here, we present a portable sequencing method using the MinION. *Pfhrp2* amplicons were generated from individual samples, barcoded, and pooled for sequencing. To overcome potential crosstalk between barcodes, we implemented a coverage-based threshold for *pfhrp2* deletion confirmation. Amino acid repeat types were then counted and visualized with custom Python scripts following de novo assembly. We evaluated this assay using well-characterized reference strains and 152 field isolates with and without *pfhrp2* deletions, of which 38 were also sequenced on the PacBio platform to provide a standard for comparison. Of 152 field samples, 93 surpassed the positivity threshold, and of those samples, 62/93 had a dominant *pfhrp2* repeat type. PacBio-sequenced samples with a dominant repeat-type profile from the MinION sequencing data matched the PacBio profile. This field-deployable assay can be used alone for surveilling *pfhrp2* diversity or as a sequencing-based addition to the World Health Organization’s existing deletion surveillance protocol.

## Introduction

Malaria rapid diagnostic tests (RDTs) offer a relatively cheap, fast, and simple alternative to traditional diagnostic microscopy. The use of RDTs led to an increase in testing of suspected malaria cases from 36 to 84% in Sub-Saharan Africa from 2013 to 2018^1^. RDTs detecting the histidine rich protein 2 (HRP2) are more sensitive and generally more heat-stable than alternative tests for *Plasmodium falciparum*^2^. *P. falciparum* accounts for over 90% of malaria cases in sub-Saharan Africa, the region with the highest malaria burden. HRP2-detecting RDTs thus constitute the majority of RDTs sold each year^3^. However, in 2010, parasites lacking *pfhrp2* (the encoding gene for HRP2) were identified in Peru^4^, indicating a threat to the efficacy of HRP2-based RDTs. Since then, numerous regional surveys have been conducted assessing the prevalence of *pfhrp2* and *pfhrp3* deletions, particularly in regions that heavily depend on HRP2 RDTs for malaria diagnosis. HRP3 is a paralog to HRP2, and can cross-react with HRP2-based RDTs, at high parasitemia levels of > 1000 parasites/mL^5^. The World Health Organization (WHO) now includes *pfhrp2/3* deletions in its list of biological threats to malaria control and tracks regional deletion prevalence surveys in its global threat map^6^. In addition, the WHO provided the “Master protocol for surveillance of *pfhrp2/3* deletions and biobanking to support future research” as a formal mechanism by which municipalities can assess the local prevalence of *pfhrp2/3*-deleted *P. falciparum*^2^*.* If this prevalence surpasses 5% in the population of clinical cases studied, replacing the HRP2 RDT is recommended. Simulation and empirical studies have both suggested that while the use of HRP2 RDTs cannot be solely responsible for the spread of *pfhrp2* deletions, their continued use does exert some selective pressure in favor of deletions (as they mask malaria diagnosis and therefore limit access to or speed of treatment) and would ultimately lead to the deletion’s fixation in the population^7,8^. An immediate global transition away from HRP2-based RDTs, however, is neither logistically feasible nor necessary in regions where *pfhrp2* deletions have not yet emerged. Public health decisions regarding the continuation or cessation of HRP2-based RDT use must be made at the regional level in response to regional prevalence.

Methods used to assess *pfhrp2* deletion and diversity have included antigen detection^9^, nested PCR^10,11^, digital droplet PCR^12^, amplicon sequencing^13^, high-throughput whole genome sequencing, and high-throughput targeted deep sequencing^7^. In many studies two or more methods are used in conjunction. While PCR may allow for indirect forms of genotyping, sequencing methods provide direct access to nucleotide content. Genotyping is an important addition to the *pfhrp2* investigation toolkit, as we currently know little about the diversity and evolution of *pfhrp2.* This is important for understanding more about how the gene operates on a population level and potentially drawing direct connections between RDT results and genotype more deeply than presence/absence. However, there are numerous limitations to overcome when applying sequencing methods to *pfhrp2* deletion and diversity surveillance.

Sanger sequencing, while not as costly as its next-generation counterparts, is a low-throughput technology that can only yield the dominant sequence in what may be a poly-clonal infection. Next-generation sequencing on Illumina platforms addresses this issue but is subject to biases for sequences with GC-content outside of the 45–65% range^14^ and produce short reads (75 to 300 bp). Exon 1 and the amino acid (AA) repeat region of exon 2 sit at the cusp of this range at 45% GC-content, and the *pfhrp2* gene has long stretches of AA repeat regions making the assembly of the approximately 1,000 base pair gene complicated using short-read sequencing methods. Targeted molecular inversion probe (MIP) deep sequencing supported by selective whole genome sequencing on the Illumina platform was used to reconstruct genomic windows surrounding *pfhrp2* and *pfhrp3* to identify deletion breakpoints, though this study did not provide repeat-type profiling (the dominant measure of *pfhrp2* variation currently), and the genotyping resolution is unclear^7^. An intuitive solution to the pitfalls of short-read sequencing for reconstructing genotypes at the nucleotide level is long-read sequencing as provided by Oxford Nanopore and PacBio sequencing platforms. In addition, Sanger, Illumina, and PacBio sequencers require generous lab space, large initial investment, and on-site expertise for device maintenance and usage. The availability of these sequencing platforms in malaria-endemic countries can thus be sparse and access to necessary sequencing reagents limited, requiring dried blood spots to be shipped abroad for sequencing.

Here, we present a portable long-read sequencing assay for assessing *pfhrp2* deletion and diversity on the MinION sequencing device. Long-read sequencing of amplicon reads largely mitigates issues surrounding de novo assembly that arise from short-read sequencing, and the MinION platform produces high data volumes which are roughly scalable by the researcher in their choice of flow cell and sequencing run duration. The capital investment is minimal in comparison to other sequencing platforms. Starter packs for the MinION Mk1B and Mk1C (a self-contained sequencing and computing device that eliminates the Mk1B’s need for a laptop) are 1,000 USD and 4,900 USD respectively as of this writing. Although the assay involves MinION-specific library preparation and flow cell loading protocols, it also eliminates the need for an expert to be present at each study site, as the operator can travel with the sequencing device and both the Mk1B and Mk1C can be easily transported in a backpack. Along with the advantages of this assay, we encountered difficulties concerning the compatibility of the *pfhrp2* target sequence with the Oxford Nanopore Technologies (ONT) barcoding kit we selected at the inception of this project. In doing so, we expose general vulnerabilities of assays based on developing technologies and highlight important considerations for future MinION assay development. Understanding and mitigating these difficulties, we propose this assay can potentially serve as an addition to the *pfhrp2* surveillance toolkit that can be incorporated into the WHO *pfhrp2/3* surveillance protocol^2^ or utilized for genetic investigations of *pfhrp2* diversity and evolution.

## Methods

### Samples

The *pfhrp2* MinION assay was developed using well-characterized reference isolates cultured at the US Centers for Disease Control and Prevention (CDC). 7G8 (Brazil), 3D7 (suspected origin Africa), HB3 (Honduras), D6 (Sierra Leone), and FC27 (Papua New Guinea), which contain *pfhrp2*, were used as proxies for positive samples, and Dd2 (Indo-China), which lacks *pfhrp2*, was used as a negative control.

The assay was evaluated using three additional sample sets. The first set was a collection of cultured control isolates from the Malaria Research and Reference Reagent Resource Center (MR4)^15^ including 7G8, 3D7, HB3, FC27, and Dd2. The MR4 set was utilized for final quality control experiments prior to sequencing field samples. Remaining DNA isolate from dried blood spot samples collected during Sub-Saharan Africa therapeutic efficacy studies (TES) that were used for opportunistic *pfhrp2* surveillance previously^9,16^ were also used. Additionally, numerous domestic and international DNA isolates were utilized, derived from whole blood and dried blood spots, respectively. International samples were collected from individuals diagnosed with malaria by microscopy. Domestic samples represent suspected cases of imported malaria from travelers seeking care at a U.S. medical facility, confirmed with RT-PCR. Whole blood samples were obtained from U.S. domestic, imported malaria cases from the CDC domestic malaria surveillance network and tested in accordance with protocol 2017-309 approved by the Office of the Associate Director of Science, Center for Global Health, Centers for Disease Control and Prevention as a surveillance activity. Informed consent was obtained from all participants. These samples were previously used to evaluate the one-step *pfhrp2* PCR protocol^17^. A subset of the clinical samples was selected to perform PacBio sequencing against which to compare the resulting *pfhrp2* types of this assay. This subset of field samples will be referred to as the PacBio set from this point forward. See Supplementary Table 1 for field sample metadata.

### One-step PCR for pfhrp2

Here, we utilized the one-step PCR protocol for *pfhrp2*^17^. A 50 µL reaction was prepared consisting of 0.126 µM each of the one-step *pfhrp2* forward and reverse primers (Supplementary Table 2), 1 × Q5 reaction buffer (New England Biolabs, MA, USA), 0.2 mM dNTPs (New England Biolabs, MA, USA), 0.02 U/µL Q5 high-fidelity polymerase (New England Biolabs, MA, USA), and 5 µL of DNA template. Each reaction batch included a positive control (7G8 or 3D7), negative control (Dd2), and nuclease-free water negative control. PCR conditions were an initial denaturation at 98 °C for 3 min (mins), a cycle of (98 °C for 30 s (s), 60 °C for 90 s, and 68° for 2 min) repeated 30 times, and a final elongation at 68 °C for 5 min. The PCR product was visualized on a 1.0% TBE gel.

### Barcoding primer design

We designed barcoding primers based on the PCR Barcoding (96) Amplicons (PBC096) kit and protocol from Oxford Nanopore Technologies (ONT) (Oxford, UK), customized for *pfhrp2* (Supplementary Table 2). Primers for barcode sequences 1–60 were assessed in silico for self- and cross-dimers. Twenty-eight barcoding primers were suitable (Supplementary Table 2). We utilized this target-specific barcoding primer method instead of ONT’s formal PBC096 protocol due to *pfhrp2-*specific issues we encountered with the kit (see Supplementary Information, Supplementary Figs. 1 and 2).

### Barcoding PCR

Barcodes were assigned to samples such that no single sequencing pool would share two samples with the same barcode. The barcoding reaction was like the one-step *pfhrp2* PCR reaction, except each sample received 0.126 µM each of the forward and reverse barcoding primer assigned to it and 10 µL of *pfhrp2* amplicon from the one-step reaction were added. PCR conditions were an initial denaturation at 98 °C for 3 min, a cycle of (98 °C for 30 s, 69 °C for 90 s, 68 °C for 2 min) repeated 30 times, and a final elongation at 68 °C for 5 min. Each reaction batch included the positive control (7G8 or 3D7), negative control (Dd2), and nuclease-free water negative control from the previous reaction. The PCR product was visualized on a 1.0% TBE gel.

### Sample pooling and library preparation

Samples were quantified on a Qubit 4 Fluorometer with the dsDNA High Sensitivity assay (Thermo Fisher Scientific, MA, USA) following the barcoding PCR and prior to pooling, using 1 µL of product. These measurements were used for experiments with normalized pools (Supplementary Information).

Pools were then purified using AMPure XP beads (Beckman Coulter, IN, USA). The beads were added to each pool at a ratio of 0.5. Library preparation for MinION sequencing was performed according to the PCR Barcoding amplicons (96) ONT protocol section “DNA repair and end-prep” followed by “Adapter ligation and sequencing cleanup” with reagents from the ONT ligation sequencing kit (SQK-LSK109) (Oxford, UK), NEBNext FFPE DNA Repair Mix (New England Biolabs, MA, USA), NEBNext Ultra II End repair / dA-tailing Module (New England Biolabs, MA, USA), and NEBNext Quick Ligation Module (New England Biolabs, MA, USA). One microliter from the final eluate of each of these two final steps was taken for quantitation on the Qubit (Thermo Fisher Scientific, MA, USA).

### MinION sequencing

Sequencing was performed on MinION Mk1B and Mk1C devices on either standard or Flongle R.9.4.1 flow cells (Oxford Nanopore Technologies, Oxford, UK). For standard flow cells the Flow Cell Priming Kit was used, and for Flongle flow cells the Flongle Sequencing Expansion Kit was used. Flow cell priming and loading was performed according to manufacturer instructions. In ONT’s MinKNOW software, barcoding was turned on, the kit was set to EXP-PBC096, and the minimum barcoding quality was set to 60. The minimum read quality score was set to 7, and no minimum read length filter was set. Base-calling and barcoding was performed by guppy v. 5.0.16 within MinKNOW. Information on sequencing run lengths can be found in the Supplementary Information (Supplementary Table 3).

### PacBio sequencing

Amplicons from the one-step *pfhrp2* reaction (Section “One-step PCR for pfhrp2”) were purified with AMPure XP beads (Beckman Coulter, IN, USA). Libraries were prepared using the 5 kb PacBio protocol with the DNA preparation kit (Pacific Biosciences, CA, USA). Samples were pooled together with 100 ng per sample and 8 samples per pool. Finished libraries were bound to P6 polymerase and sequenced on the PacBio RSII with C4 chemistry with one pool per SMRTcell.

### Analytical pipeline

Quality filtered reads were aligned to exon 1 and the repeat region of exon 2 of the *pfhrp2* reference sequence (PF3D7_0831800) with minimap2 (v. 2.21-r1071) using its “map-ont” option^18,19^. The SAM file was sorted and converted to a BAM file with samtools sort (v. 1.13), and the BAM file was converted to a FASTQ file using samtools fastq^20^. Mapping statistics were calculated using Qualimap (v. 2.2.2)^21^ and compiled into reports with MultiQC (v. 1.10.1)^22^. The FASTQ containing successfully aligned sequencing reads was then used as input for de novo assembly with canu (v. 2.1.1)^23^ using its “-nanopore” option and “genomeSize” 1.3 kb. We used aligned reads for assembly to eliminate the inclusion of off target reads. De novo assembly was also performed in Geneious 2022.0.2. The canu and Geneious contigs were all converted to consensus sequences, visually explored, and manually edited to fit in the correct translation frame if necessary, based on AA marker sequences (Table 1, Supplementary Information). Up to ten contig consensus sequences per sample were curated based on their having > 10 reads and few low-quality bases. The nucleotide sequences were then translated to AA sequences and exported to FASTA format. Repeat type counts were calculated by a Python (v. 3.8.5) script (Data and Code Availability). Visualizations of repeat-type counts were created using the Python library seaborn (v. 0.11.2)^24^ in Jupyter Notebooks^25^. For PacBio-sequenced data, an analogous analytical pipeline was used, with minimap2’s “map-pb” option and otherwise identical treatment. Analysis was performed using a MacBook Pro 2016, 2.4Ghz i9 and 32 GB DDR4, running MacOS Monterey Version 12.4.

**Table 1:** Amino acid sequence markers for *pfhrp2*.

Marker	Amino acid sequence
Exon 1	MVSFSKNKVLSAAVFASVLLDN
Exon2 start	NNSAFNNNLCSKNA
Beginning of repeat region of exon 2	VDD
End of repeat region of exon 2	CLRH
**Repeat types**	
Type 1	AHHAHHVAD
Type 2	AHHAHHAAD
Type 3	AHHAHHAAY
Type 4	AHH
Type 5	AHHAHHASD
Type 6	AHHATD
Type 7	AHHAAD
Type 8	AHHAAY
Type 9	AAY
Type 10	AHHAAAHHATD
Type 11	AHN
Type 12	AHHAAAHHEAATH
Type 13	AHHASD
Type 14	AHHAHHATD
Type 30	AHHAVD
Type 31	SHHAAY

These markers were used as guide for correctly framing the *pfhrp2* sequences for translation. Not all repeat types are present in all positive samples. The region between amino acid sequences VDD and CLRH was considered the repeat region of exon 2 and inspected for repeat types.

### Kelch 13 control experiment for barcoding method

To determine if uneven performance between barcodes with normalized DNA input was an issue specific to *pfhrp2*, we designed a version of our assay for *k13* amplicons. Five barcoding primer pairs were designed for *k13* and checked for self- and cross-dimers in silico prior to being ordered (Supplementary Table 4). *k13* was amplified from a panel of reference strains including 7G8, 3D7, HB3, D6, and Dd2 according to the Malaria Resistance Surveillance Project (MaRS) protocol^26^. The amplicons were then barcoded with *k13*-specific barcoding primers under the same PCR conditions described in Section “Barcoding PCR”. Two normalized sequencing pools were made, the first containing the reference panel and the second containing 7G8 replicates. Bead purification, library preparation, and sequencing were performed as described in Section “MinION sequencing”.

### Barcode primer in vitro experiment

Given uneven performance between barcodes, we assessed the performance of each on normalized sequencing pools of 7G8 replicates to determine which performed better than others and should therefore be more heavily utilized. The 28 barcodes were spread across 6 sequencing pools with 3–5 samples each and sequenced on standard flow cells for 3 h. The following ten barcodes were selected for the evaluation experiments: bc01, bc02, bc12, bc13, bc17, bc18, bc49, bc30, bc52, and bc60 (Supplementary Information, Supplementary Table 5).

### Evaluation

**Figure 1:**
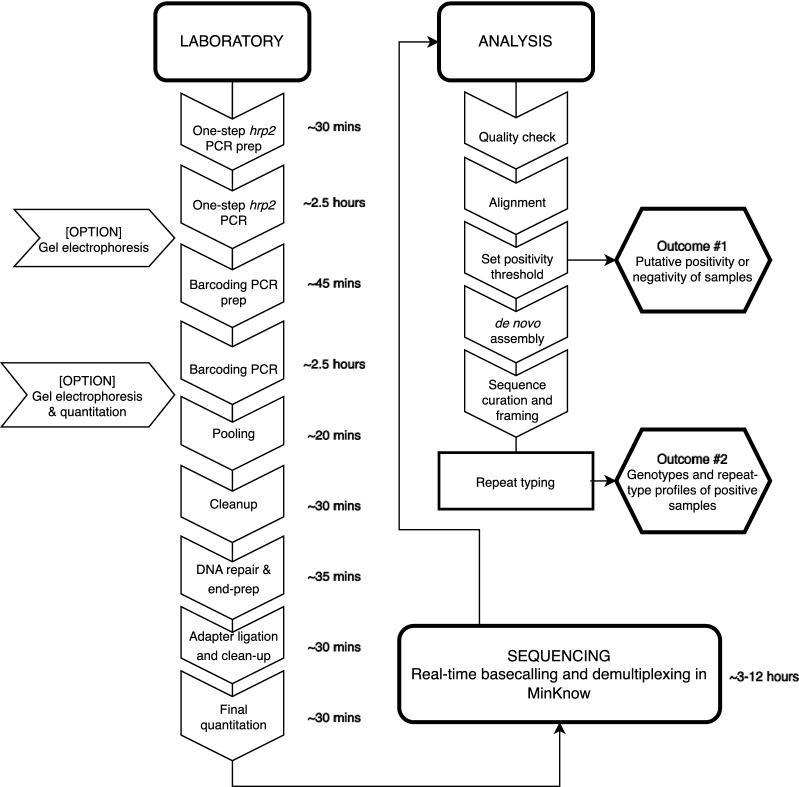
Workflow for field sample processing and analysis with the minION *pfhrp2* assay. The optional steps of gel electrophoresis and pre-pooling quantitation were performed for quality control purposes. Time estimates are based on the researcher’s experience preparing a single pool of 5–10 samples, including a positive and negative control. See “Methods” for information on the processing of a subset of field samples for PacBio sequencing.

The MR4 and PacBio sets were normalized, and the remaining field samples were not, given the expectation in field-based use of this assay that there will be *pfhrp2* positive and negative samples. Otherwise, samples used for evaluation were treated identically according to the methods described in Sections “One-step PCR for pfhrp2” to “MinION sequencing”. To confirm *pfhrp2* negativity and to assess the viability of a sample for genotyping, we implemented a coverage-based positivity threshold (Supplementary Information, Supplementary Table 6). In a field application, the results of this threshold test are the first of two possible outcomes from this assay (Fig. 1). Putatively positive samples from this step were processed for repeat-typing as described in Section “Analytical pipeline”.

## Results

### Pfhrp2 inhibits equal performance of individual PCR barcodes from Oxford nanopore

**Figure 2:**
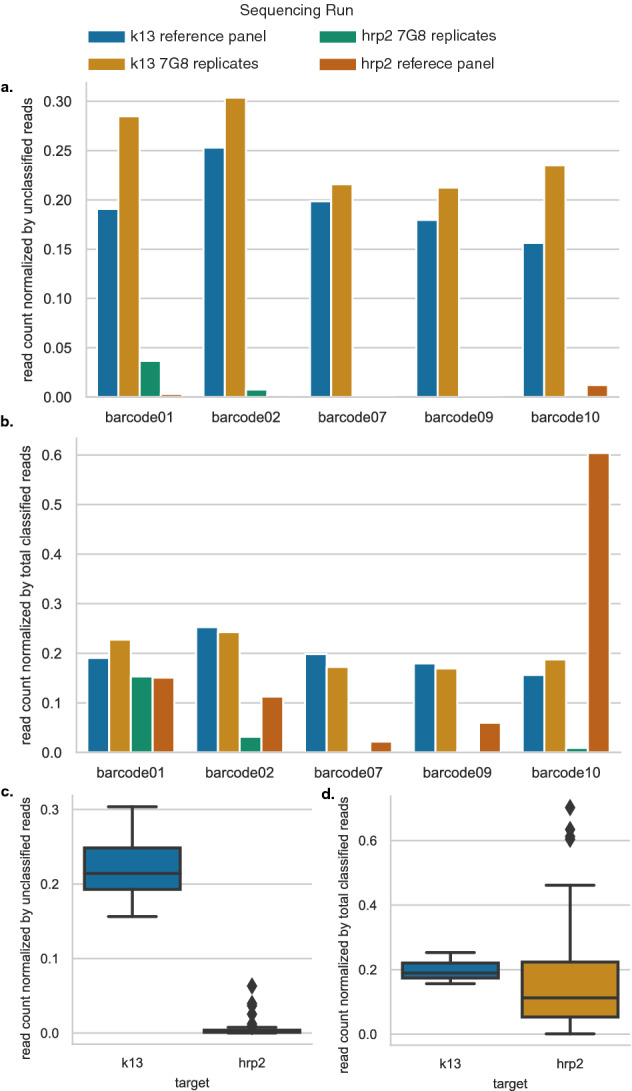
Differences between *k13* and *pfhrp2* barcode performances with the custom-barcoding protocol. (**a**) Barcode performance across two *k13* and two *pfhrp2* control sequencing runs, between five barcodes, as measured by barcode read count normalized against the amount of unclassified (not barcoded or properly de-multiplexed reads) reads in the sequencing run. One sequencing run for each target was performed on a diverse reference panel (see blue and green bars) and replicates of control strain 7G8 (see orange and red bars). (**b**) Read counts normalized by total passing reads across two *k13* and two *pfhrp2* control sequencing runs, between five barcodes. (**c**) Summarized barcode performance (barcode read counts normalized by unclassified reads) across multiple sequencing runs for *k13* (n = 2) and *pfhrp2* (total n = 9; reference panel sequencing runs, n = 3; barcode performance test, n = 6). (**d**) Summarized barcode performance (barcode read counts normalized by total classified reads) across multiple sequencing runs for *k13* (n = 2) and *pfhrp2* (total n = 9; reference panel sequencing runs, n = 3; barcode performance test, n = 6).

To further explore the uneven performance of barcodes across normalized control DNA input, we prepared and sequenced two pools of barcoded *k13* amplicon to compare against the *pfhrp2* results. One pool contained five different reference strains (see “Methods”) and one pool contained replicates of 7G8. Barcodes across both the *k13* pools performed better than their counterparts in comparable *pfhrp2* control pools when sequenced for the same amount of time with pools containing the same number of different samples (Fig. 2). Read counts across barcodes were more even for *k13* than for *pfhrp2* (Supplementary Fig. 5). In the *k13* experiments, the correctly classified reads performed consistently better against the quantity of “unclassified” reads (Fig. 2c). The proportion of correctly classified reads to total classified reads would be expected to hover around 0.20 for a 5-sample sequencing pool, and for *k13*, this was the case. Barcodes for the *pfhrp2* experiments performed stochastically, spanning from near 0 to 0.8 (Fig. 2d). These differences in barcode performance across comparable sequencing pools and identical workflows suggests that *pfhrp2* created difficulty either for the sequencer or demultiplexing program when used with the PBC096 barcoding kit.

### Assessing pfhrp2 negativity with MinION sequencing

**Figure 3:**
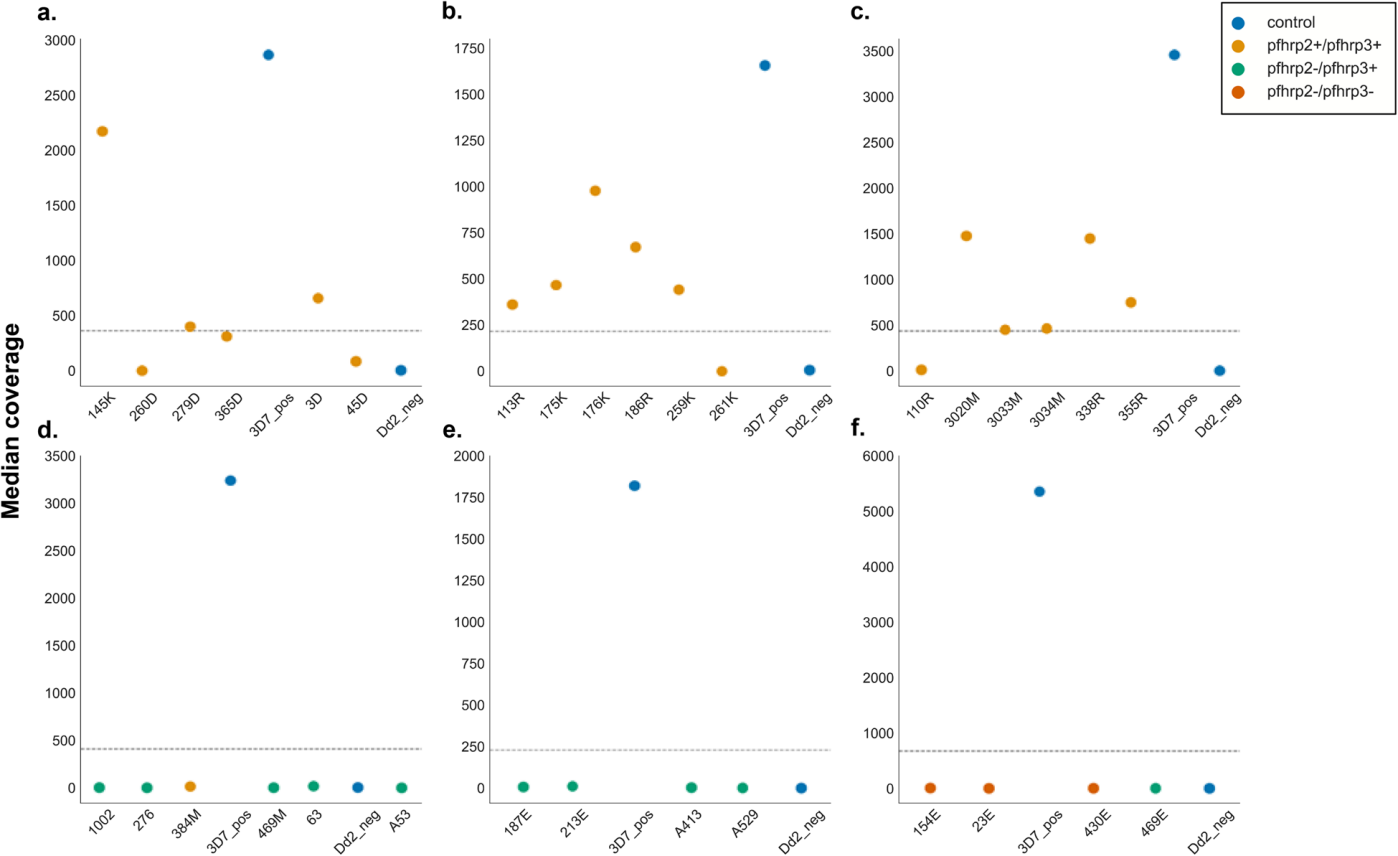
Positivity thresholds for previously analyzed TES sample dataset. Based on the positivity threshold established for each sequencing pool, calculated as the first octile between the median coverage of the Dd2 negative control and 3D7 positive control, largely reifies the findings from a previous analysis of these samples^9,16^. Notably, no samples previously assessed to be *pfhrp2-* were found to be putatively positive using this MinION assay. Positivity thresholds are shown here for sequencing runs (**a**) FL1_TES, (**b**) FL2_TES, (**c**) FL3_TES, (**d**) FL4_TES, (**e**) FL5_TES, and (**f**) FL6_TES.

Crosstalk between barcodes creates the issue of *pfhrp2* negative samples possibly having non-zero coverage when aligned to the reference sequence. We witnessed this phenomenon with our Dd2 negative control, which was sequenced with each field sample pool and found that median coverage on *pfhrp2* in the negative controls scaled roughly with the total passing reads yielded by the sequencing run (Supplementary Fig. 3, Supplementary Tables 3 and 6). Barcode crosstalk in nanopore sequencing has been previously reported elsewhere^27^. However, the positivity threshold we present here (see Methods) was intended to mitigate false identification of *pfhrp2* positive samples due to barcode crosstalk. This method successfully confirmed all *pfhrp2* negative samples that were included in the TES sample set, for which *pfhrp2* positivity and negativity had been previously established by antigen test and PCR (Fig. 3). Importantly, many *pfhrp2* positive samples in this set did not surpass the threshold (Fig. 3). As the TES pools were not normalized, this could be due to the quantity of DNA added, though the stochastic performance of barcodes in this assay hinders our ability to present an exact cause. This issue of potential false negatives (i.e., positive samples with low coverage) highlights the importance of using this assay as an additive and confirmatory companion to inferring *pfhrp2* negativity through other methods. However, it also eliminates low coverage and potentially crosstalk-impacted samples from the genotyping stage, minimizing the likelihood of calling *pfhrp2* repeat types based on crosstalk. A total of 33/38, 13/32, and 47/82 samples were called as positive for the TES, PacBio, and remaining field sample set, respectively (93/152 total field samples) (Fig. 3, Supplementary Fig. 6).

### MinION assay supports pfhrp2 repeat typing

**Figure 4:**
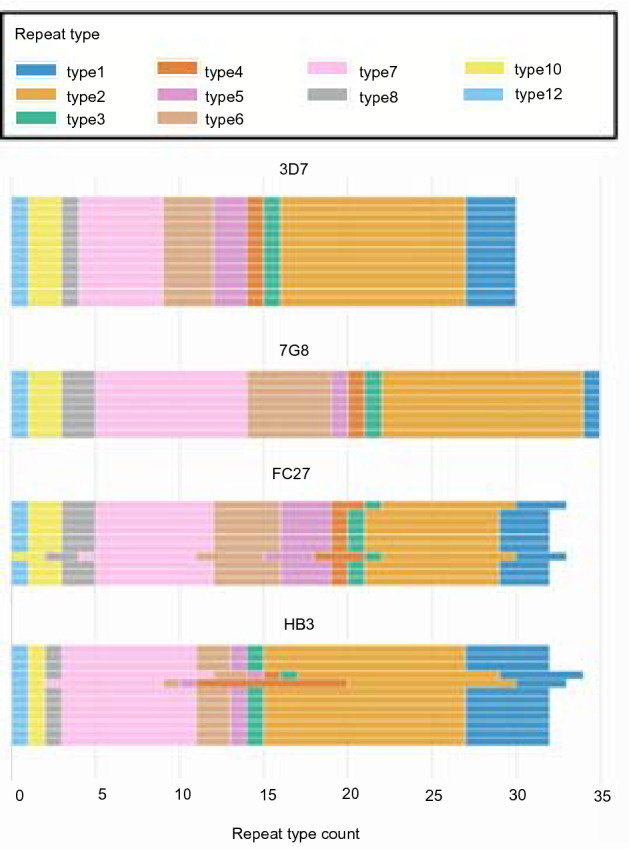
Repeat-type profiles from reference strain experiments. Each colored bar indicates the count of a particular repeat type in exon 2 of *pfhrp2* as color-coded in the legend. Each row represents a single contig constructed based on de novo assembly performed on reads that successfully aligned to the *pfhrp2* 3D7 reference sequence. Due to the repetitive nature of exon 2, minor variations in contig construction, as seen here for FC27 and HB3, can be expected, even in monoclonal isolates.

**Figure 5:**
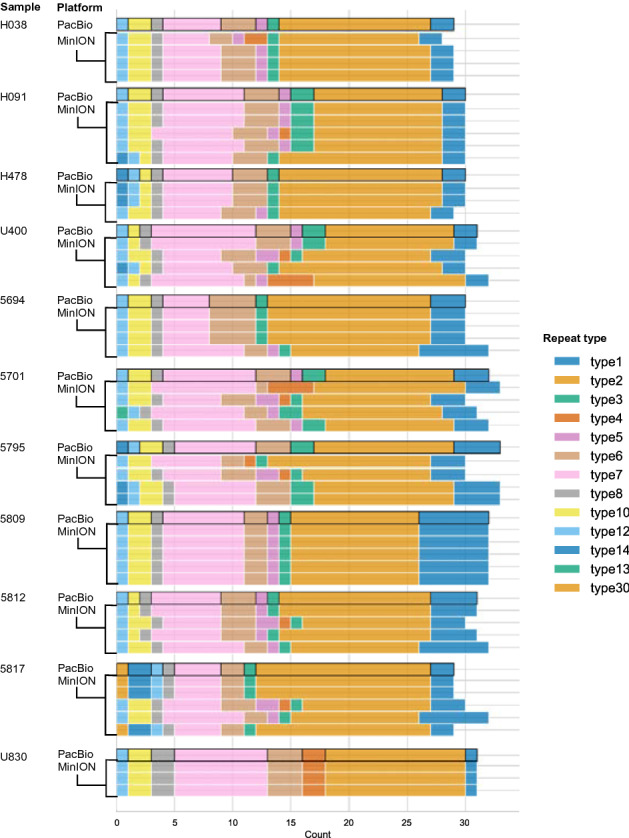
PacBio and MinION contig repeat type patterns for field samples. A random selection of contig repeat-type patterns from the PacBio sample set, comparing the patterns in the PacBio-derived contig with the MinION-derived contigs. For all repeat-type patterns, see Supplementary Table 7.

We were able to successfully type four well-characterized reference strains across four sequencing runs with this assay (Fig. 4, Supplementary Table 7). For the PacBio set, we compared contigs generated with PacBio sequencing to those generated with MinION sequencing. Of 32 putatively positive samples identified using the positivity threshold, 31 had a minimum of one contig in agreement with the PacBio sequence. Eighteen of the samples from the PacBio set had a clear dominant type (≥ 50% of typed contigs), and all dominant repeat types but one matched that of its PacBio-sequenced counterpart (Fig. 5, Supplementary Table 7). This sample, 8d, had a majority repeat type with three out of five curated contigs in agreement, but had one contig that did match the PacBio contig. Two samples had no MinION repeat types that matched the PacBio contig. The TES sample set had 13 *pfhrp2* positive samples according to the octile threshold, with 11 of these having a clear majority repeat type. Six of those samples had 100% agreement across contigs. One sample had agreement between 2 out of 10 contigs produced, and one sample, 259 K, had no agreement between 4 contigs. For the remaining 47 positive field samples, 33 had a clearly dominant repeat type and an additional 5 had agreement between 2 or more contigs that did not reach 50%. Considering all putatively positive field samples, 62/93 had a dominant repeat type. Additionally, we successfully reconstructed the positive control repeat types (either 3D7 or 7G8) in each sequencing run.

## Discussion

### The *MinION* assay for deletion detection or genetic characterization for *pfhrp2* provides logistical flexibility for enhancing *pfhrp2* surveillance activities

With the recent increase in *pfhrp2* deletions in malaria endemic countries, having a portable sequencing assay for confirmation of *pfhrp2* is becoming more critical for routine molecular surveillance. *Pfhrp2* deletion threatens the efficacy of the most widely used RDT in Sub-Saharan Africa. Outright replacement of HRP2 RDTs is impractical, but regional substitution of HRP2 RDTs in favor of alternatives is necessary to achieve malaria control in regions with high prevalence of *pfhrp2* deleted parasites.

The MinION *pfhrp2* assay presented here would allow scientists and healthcare professionals to genetically confirm *pfhrp2* deletion status and characterize *pfhrp2* diversity among non-deleted samples at a location of their choice by eliminating the need for bulkier, more expensive sequencers and potentially sample transport. A primary benefit of the MinION platform for this application is the flexibility it provides to be used at primary sample collection sites or centralized laboratories, neither of which would require established, permanent genomics laboratories. The use of this assay in conjunction with a portable PCR device such as miniPCR (miniPCR bio, MA, USA) could ease the logistical burden of sample transportation for a lower cost than outfitting regional facilities in malaria-endemic areas with in-house PCR and sequencing systems and expertise. This assay can be performed at any laboratory facility with cold-chain storage and the equipment required for DNA extraction from whole blood or dried blood spots. The sequencer can be transported by backpack. While this portability does not eliminate the need for sample transport entirely (*e.g.,* in surveys including dozens of collection sites), it increases the speed of data generation, allows flexibility in experimental design and logistics and can reduce regional dependence on international partners to run *pfhrp2* surveillance programs that include a sequencing component.

In addition to this assay’s utility in accompaniment to the WHO protocol for *pfhrp2/3* surveillance, it may be used for general research on *pfhrp2* deletions and diversity outside the protocol. The portability of the assay increases geographic and temporal flexibility for research investigating the evolution of this gene. When used within or outside of the WHO protocol for *pfhrp2* deletion surveillance, data generated using this assay and shared could be used additively for broad surveillance of *pfhrp2* diversity. As of this writing, the public health community understands little about why HRP2 is non-essential. Researchers have roughly calculated the fitness costs of *pfhrp2/3* deletion in vitro to be on par with drug resistance mutations^28,29^. Additionally, further insights on how *pfhrp2* performs on a population level and how it has emerged require broad geographical and temporal sampling accompanied by sequencing. Akinyi and colleagues found there to be multiple genetic origins of the *pfhrp2* deletion in a *P. falciparum* sample set from Peru, the country where the *pfhrp2* deletion was first identified^11^. Furthermore, the diversity and prevalence of deletion types, whether they include exons 1 and 2, only exon 1, or only exon 2, remains unclear. Understanding how genetic variants of *pfhrp2* may impact RDT performance will be another important area of research that has, to our knowledge, only been explored indirectly through limit of detection evaluations of different RDT products^30^.

Here, we generated sequencing data on two R.9.4.1 flow cells available from ONT: the standard flow cell (up to 900 USD per flow cell) and the Flongle (~ 90 USD per flow cell). The optimal flow cell depends on the volume of samples being pooled for a single sequencing run and the desired speed of data recovery. The advantage of the standard flow cell is its capacity and longevity compared to the Flongle. Standard flow cells are considered in good condition with a minimum of 800 active sequencing pores and are under warranty for 3 months after purchase, whereas Flongles perform with approximately 10% of the pores and are under warranty for 1 month. When processing 8 or more samples in parallel with an allowance of 12 h for sequencing and basecalling/demultiplexing or when processing 5 or more samples with an allowance of 6 h for sequencing and basecalling/demultiplexing, the standard flow cell would offer superior data. However, if there is a greater time allowance, with up to 24 h for sequencing and demultiplexing for pools of 6–8 samples, the Flongle would offer a lower cost per sample.

### Limitations of current study

The foremost limitation of this assay lies in its quantitative ambiguity. We found there to be *pfhrp2*-specific issues that affected the consistency and degree of barcode performance regardless of whether the pooled DNA was normalized or not.

Another limitation lies in the quality of sequencing reads and the impact of low quality on the de novo assemblies. The relatively low quality of reads from R.9.4.1 flow cells and the repetitiveness of *pfhrp2* may cause difficulty in resolving assemblies. The unpredictable yield of each barcode exacerbates this issue, as it is difficult to distinguish whether assemblies from single samples with multiple singleton repeat type patterns are a result of sample quality and quantity of input DNA or of poor barcode performance. Though we do not anticipate singleton repeat-type patterns to reflect true intra-sample diversity (i.e., evidence of a polyclonal infection), we have not assessed the performance of this assay with known poly-clonal samples.

The read quality limitation may be short-lived due to the release of enhanced chemistry from ONT, including R.10 flow cells and a new version of the Ligation Sequencing Kit used here. We do not anticipate changes to the workflow considering this development, but we do anticipate better resolution in the resulting de novo assemblies. Since the inception of this project, ONT has released numerous alternative kits, protocols, and software versions that were not explored as part of this project. Rapid development within the fields of long-read sequencing, portable laboratory systems, and data processing are, while ultimately advantageous, a salient challenge in the development and validation of stable genetics assays for applications to public health.

Though not a limitation of the method described here, our evaluation did not include any exploration of the *pfhrp2* diversity found in the field samples. We did not develop a sample pool for the sake of this project with power to determine longitudinal or spatial nucleotide diversity or repeat-type profile diversity.

Lastly, this assay inherits the limitations of the one-step *pfhrp2* PCR method presented by Jones et al.^17^. In brief, the size of *pfhrp2* deletions that may surpass exons 1 and 2 cannot be characterized here due to the size of the amplicon, variables such as sample type and PCR efficiency obfuscate the limit of detection of the PCR method and therefore the sequencing method, and this assay does not address *pfhrp3*^17^. Cross-reactivity between HRP2 and HRP3 in HRP2-based RDTs was recently proposed^5^, but it remains unclear how deletions in one or both genes affect clinical sensitivity of HRP2-based RDTs.

As noted in the introduction, there are numerous methods recently proposed for enhancing investigations of *pfhrp2* deletion and diversity, including high-resolution targeted and whole genome sequencing. The assay proposed here is one of several options, with its unique set of advantages and drawbacks. Here, we present a first step towards developing a portable and analytically nimble option for confirming *pfhrp2* deletions or assessing *pfhrp2* diversity, highlighting ways in which the combination of our target sequence and sequencing chemistry complicates practical application.

### Future work

We present the *pfhrp2* MinION assay as a proof of concept, with multiple avenues for improvement and optimization. This might include the evaluation of some of the many barcoding kits offered by ONT, which may mitigate the barcoding (and therefore quantitative) issues we encountered in the development of this assay using the PCR barcoding (96) kit. A recent report found nanopore sequencing to be less error prone for AT-rich sequences in PCR-free approaches^31^. Utilizing a barcoding kit that would require no further amplification of the *pfhrp2* product as the assay does in its current state may increase the reliability and reduce the error rate in the sequencing data. Another parallel avenue of optimization could be the implementation of ONT’s less error prone R.10 chemistry, which may result in fewer errors and more reliable sequence reconstruction. Experimentation with combinations of different filtering parameters such as fragment length and minimum base-calling score is another avenue, unexplored here, that may optimize the assay’s output. Given the value of this method for exploring the population of *pfhrp2* copies that may exist within a sample, a controlled in vitro exploration of how poly-clonal samples would present analytically using R.10 chemistry is an important next step in optimizing this assay for broad genotypic surveillance of *pfhrp2* diversity.

## Supplementary Information

Supplementary Information.

Supplementary Tables.

## Data Availability

Bash and Python code used for data analysis can be found on Github at https://github.com/sjsabin/minion_hrp2_project. Sequencing summaries have been deposited on Zenodo (https://doi.org/10.5281/zenodo.6780399). We used the following as our *pfhrp2* reference sequence: NCBI Gene database PF3D7_0831800. The gene sequences produced and analyzed here have been deposited on the NCBI GenBank database, under accession numbers OP186488-OP186871. Accession numbers are listed in completion in Supplementary Table 7.
