# Supplementary material for: Portable and cost-effective genetic detection and characterization of Plasmodium falciparum hrp2 using the MinION sequencer
Source: Sci Rep. 2023 Feb 18;13:2893. doi: 10.1038/s41598-022-26935-z (PMC9938884; doi:10.1038/s41598-022-26935-z)
Supplement: Supplementary file 1 — Supplementary Information. [file 41598_2022_26935_MOESM1_ESM.pdf]

Supplementary Information for "Portable and cost-effective genetic detection and characterization of *pfhrp2* using the MinION sequencer"

Susanna Sabin[1,2*], Sophie Jones[1,3], Dhruviben Patel[1,2], Gireesh Subramaniam[1,2], Julia Kelley[1], Michael Aidoo[1], Eldin Talundzic[1]

[1]Malaria Branch, Division of Parasitic Diseases and Malaria, Center for Global Health, Centers for Disease Control and Prevention, Atlanta, Georgia, USA. [2]Oak Ridge Institute for Science and Education, USA. [3]Williams Consulting, USA.

*Address correspondence to Susanna Sabin, Malaria Branch, Division of Parasitic Diseases and Malaria, Center for Global Health, Centers for Disease Control and Prevention, Atlanta, Georgia, USA.
Email: qwg8@cdc.gov, 404-718-4440.

Comparison of Oxford Nanopore Protocol with custom barcoding protocol

In our preliminary data from our execution of the *pfhrp2* assay we found uneven distribution of read counts between different barcodes despite the normalization of input DNA, a concerning abundance of "unclassified" reads that had not been successfully assigned to a barcode, and the presence of spurious barcode assignments (*e.g.* reads assigned to barcodes that had not been used). To assess if these issues were the result of our experimental design, we chose to compare the performance of our custom-designed barcoding primer approach with ONT's PCR barcoding (96) amplicon protocol. For equivalency, we used replicates of 7G8. ONT's protocol calls for the design of a tailing primer that would amplify the target region while yielding product that would include the target amplicons plus a "tail" that would then act as the target for barcoding primers.

To test the full Oxford Nanopore Technologies (ONT) PCR barcoding (96) kit protocol we designed tailing primers specific to the *pfhrp2* sequence using the one-step PCR primers (Supplementary Table 2) (Jones, et al., 2020).

**Primer**	**Sequence (5' to 3')**
Tailing segment FORWARD	TTTCTGTTGGTGCTGATATTGC
Tailing segment REVERSE	ACTTGCCTGTCGCTCTATCTTC
hrp2 one-step FORWARD	ATGATTCATTATTCTATATTTATAAGGAAGATTAC
hrp2 one-step REVERSE	CACTTCATGTATTTATGTATGCAGAAC

We tested the *pfhrp2* tailing primers with the Thermo Fisher Multiple Primer Analyzer (Breslauer, et al., 1986). When combined with the ONT tailing primer sequence without reduction, the forward primer created a self-dimer:

Self-Dimers:

1 dimer for: seq_1

5-tttctgttggtgctgatattgcatgattcattattctatatttataaggaagattac->

| | | |||| | || || || || | |||| | | |

<-cattagaaggaatatttatatcttattacttagtacgttatagtcgtggttgtcttt-5

Due to the self-dimer, we reduced the *pfhrp2* one-step forward primer. The tailing primers we used were:

**Forward (5' to 3'):**	TTTCTGTTGGTGCTGATATTGCATGATTCATTATTCTATA
**Reverse (5' to 3'):**	ACTTGCCTGTCGCTCTATCTTCCACTTCATGTATTTATGTATG

We found the tailed primer did not successfully amplify *pfhrp2* product from raw extract but did yield product when used on pre-amplified *pfhrp2* product. The tailed product was then purified with AMPure XP beads at a ratio of 0.5x beads and carried forward to the barcoding PCR step. Pooling, cleanup, library preparation, and sequencing were then performed as described above. An initial quality check showed that the proportion of unclassified reads in relation to the correctly assigned barcodes was far lower using the ONT protocol than in control experiments using the custom barcoding method presented here. However, when the reads were aligned to the 3D7 *pfhrp2* reference sequence (PF3D7_0831800) and aligned reads were assembled using canu (2.1.1) (Koren, et al., 2017), the resulting sequences were truncated and frequently chimeric (see Supplementary Information, Supplementary Figure 2 for examples). Despite the superior performance of the ONT protocol, we were unable to assemble the data to reflect the correct length and repeat-type sequence of the 7G8 control, either by reference-based or *de novo* assembly, and thus proceeded with our original approach.

Pool Normalization

To normalize sequencing pools, the sample with the lowest concentration (excluding the Dd2 negative control when applicable) was used as the normalization target. The lowest concentration in ng/µL was multiplied by the total remaining µL of product to find the target total nanograms to add to the pool from each sample.

$$\left(Lowest\ \frac{ng}{\mu L}\right) \cdot (Total\ remaining\ \mu L) = ng\ to\ add\ to\ pool$$

For all other samples, the quantity of product to add to the pool could be calculated as follows:

$$\frac{(ng\ to\ add\ to\ pool)}{\left(sample\ \frac{ng}{(\mu L)}\right)} = \mu L\ to\ add\ to\ pool$$

Sequencing run length

The length of each sequencing run was determined by the number of samples included in the sequencing pool (Supplementary Table 3). Typically for standard flow cells, pools of 3-5 were run for 3 hours and pools of 5-10 were run for 6 hours. We did not sequence pools with more than 10 samples. For the Flongles, we found that sequencing runs longer than 12 hours plateaued at the 12-hour mark (Supplementary Figure 3). Flongle sequencing runs with 3-5 samples were set to run for 6 hours and

those with 5-8 samples were set to run for 12 hours. We did not explore sequencing more than 8 samples per Flongle.

Manual sequence editing

When opening consensus sequences from *de novo* assembly contigs in Geneious, the first two sequences we searched for were exon 1 (5' - ATGGTTTCCTTCTCAAAAAATAAAGTATTATCCGCTGCCGTTTTTGCCTCCGTACTTTTGTTAGATAAC – 3') and the start of exon 2 (5' - AATAATTCCGCATTTAATAATAACTTGTGTAGCAAAAATGCA - 3'). To simplify this process, we created an annotated version of the 3D7 *pfhrp2* reference sequence (PF3D7_0831800), which contained the following annotations:

**Annotation name**	**Nucleotide Sequence (5' to 3')**
EXON 1	ATGGTTTCCTTCTCAAAAAATAAAGTATTATCCGCTG CCGTTTTTGCCTCCGTACTTTTGTTAGATAACG
Exon2_start	AATAATTCCGCATTTAATAATAACTTGTGTAGCAAA AATGCA
Intron	CTATATAAAAATAAAAAAGCATTTAAAAATGAAAAA ATTTATATATATACAATATATAAATATATATATATAT ATATATATTATTATTATATGTATTTTTATAATTGAATG ATATATTTATTTCTATTTGCAATTAAAATGCTTAC
**Repeat types:**	
1	GCCCATCATGCTCATCATGTAGCCGAT
2	GCCCATCATGCTCATCATGCAGCCGAT
3	GCCCATCATGCTCATCATGCAGCCTAT
4	GCCCATCAT
5	GCTCATCACGCTCATCATGCATCCGAT
6	GCTCACCATGCAACCGAT
7	GCTCATCATGCAGCTGAT
8	GCTCACCATGCAGCTTAT
10	GCTCATCATGCAGCCGCACACCATGCAACTGAT
12	GCTCACCATGCAGCCGCACACCACGAAGCCGCCACA CAT

When opening a consensus sequence, we would navigate to the "Live Annotate and Predict" tab of the right-hand sidebar menu and check the "Annotate from..." box with the source as "Reference Features," the folder in which our annotated reference sequence was saved. The repeat types in 3D7 do not include the full diversity of repeat types found in *pfhrp2.* These annotations assist the researcher in correctly identifying components of interest in *pfhrp2* and correctly framing the sequences for amino acid translation (see Table 1). Exon 1 and exon 2 are in reverse orientation, and consensus sequences occasionally needed to be reversed. While exon 1 and the beginning of exon 2 are likely to be identical across *pfhrp2* positive samples, the intron is likely to vary, and may not be annotated in consensus sequences. We deleted the intron from the consensus sequences to keep many in frame. We do not expect the intron to be well resolved due to a lengthy AT repeat, and the intron generally pushes exon 2,

and therefore the repeat typing sequences, out of the correct translation frame. We inspected “N” and other ambiguous base calls by comparing the consensus sequence with the contig in the case of Geneious assemblies. In the case of canu assemblies, if there was an “N” included in the tig FASTA that pushed the sequence out of frame, we would experiment with deleting it to see if the sequence would revert to the correct translation frame. In both cases, if the frame could not be resolved, the contig was ignored. As we assessed multiple contigs per samples, we do not believe this practice excluded true diversity from our analyses.

Barcode scoring

To mitigate differences in data yield between the pools, the number of reads assigned to each barcode was divided by the total number of true classified and total classified reads. True classified reads refer to those assigned to a barcode that was included in the sequencing pool. Total classified reads refer to all reads that were classified to any barcode, whether correctly or spuriously. We found the number of spuriously assigned reads to vary stochastically between sequencing runs. The product of these two normalized values was then used to calculate the overall barcode score (Supplementary Table 5).

Positivity thresholds

In accordance with prior observations (Xu, et al., 2018), we observed a small amount of cross-talk between barcodes through the appearance of *pfhrp2* sequences in negative controls. We do not consider this to be pre-barcoding cross-contamination due to the Dd2 negative controls remaining gel negative across all experiments. Additionally, the median coverage of *pfhrp2* in Dd2 negative controls roughly scales with the total reads yielded by the sequencing run (Supplementary Figure 4, Supplementary Tables 3 and 6). We chose to mitigate this crosstalk by setting a putative positivity threshold for each sequencing run, based on the first octile between the median coverages of the negative and positive controls from the minimap2 alignment. This method was found to call all previously established *pfhrp2* negative samples from the TES set as negative. The calculation and visualization of these thresholds were performed in a python (v. 3.8.5) Jupyter Notebook (Kluyver, et al., 2016). We emphasize that this positivity threshold requires a successful positive control and a negative control that is PCR negative. If these conditions are not fulfilled, a *pfhrp2* signal in the negative control may indicate cross-contamination or laboratory error, and the threshold would be meaningless for establishing a boundary pertaining to crosstalk.

Figures

a.

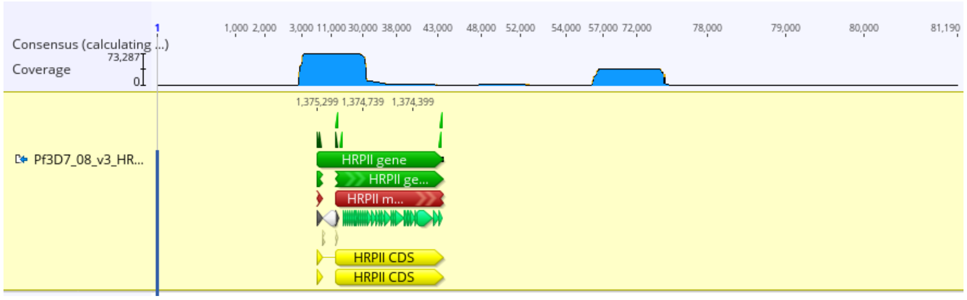

b.

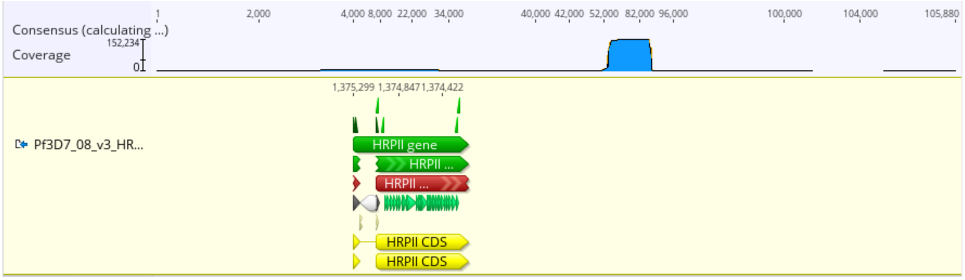

c.

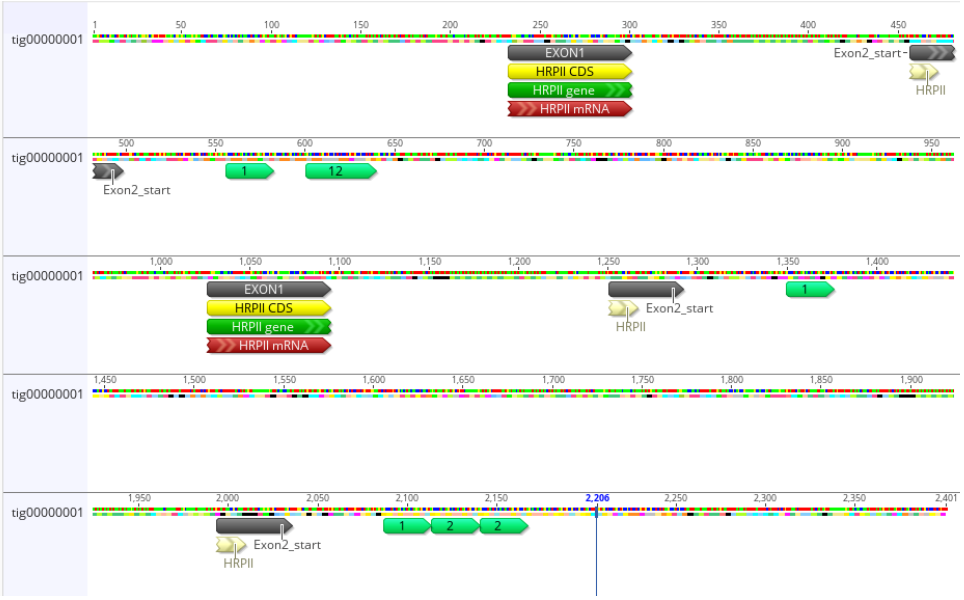

d.

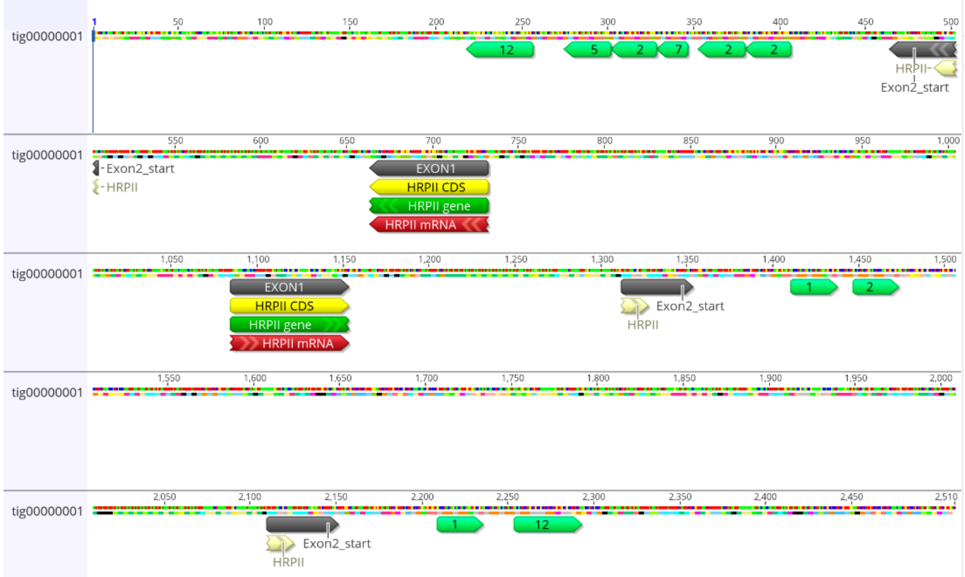

Supplementary Figure 1. Using the ONT protocol yielded truncated or chimeric reads, and examples of this are visualized here as screenshots from Geneious. When aligned to the PF3D7_0831800 sequence,

the distribution of read coverage clustered outside of the target sequence or on the first half of the target sequence. A) Aligned sequencing reads from sequencing run FC1, barcode01. B) Aligned sequencing reads from sequencing run FC1, barcode02. C) Canu contig from FC1, barcode01 showing truncated assembly (see the jump from repeat type 1 to repeat type 12). D) Canu contig from FL2, barcode01 showing chimeric assembly (note the position and direction of the exon 1 annotations).

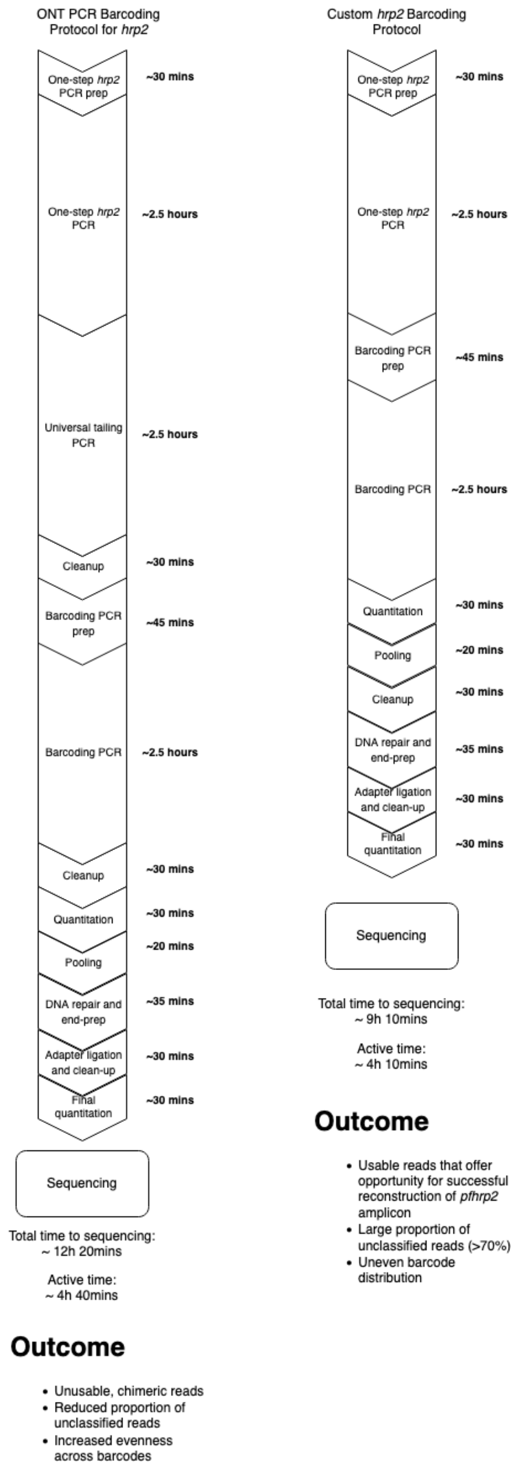

Supplementary Figure 2. Comparison of ONT and custom *pfhrp2* MinION sequencing workflow.

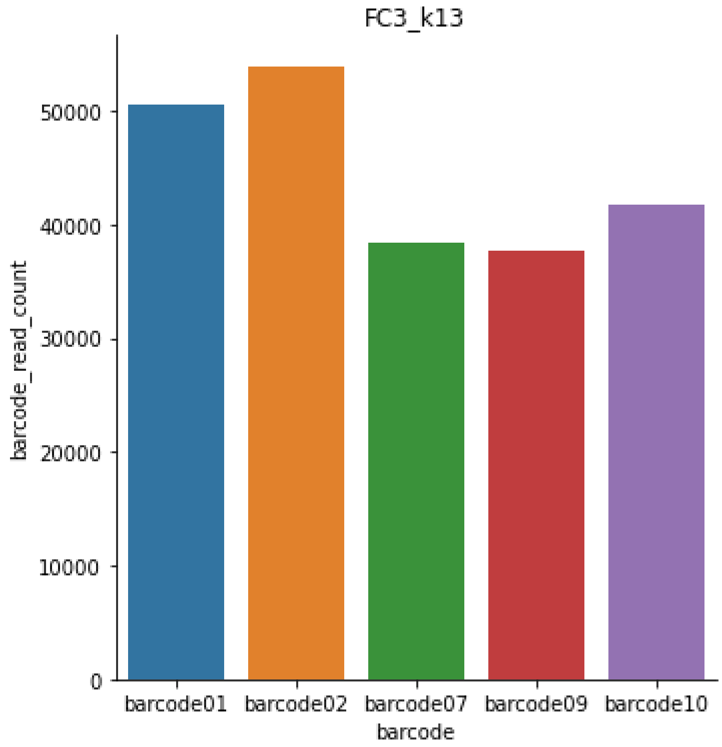

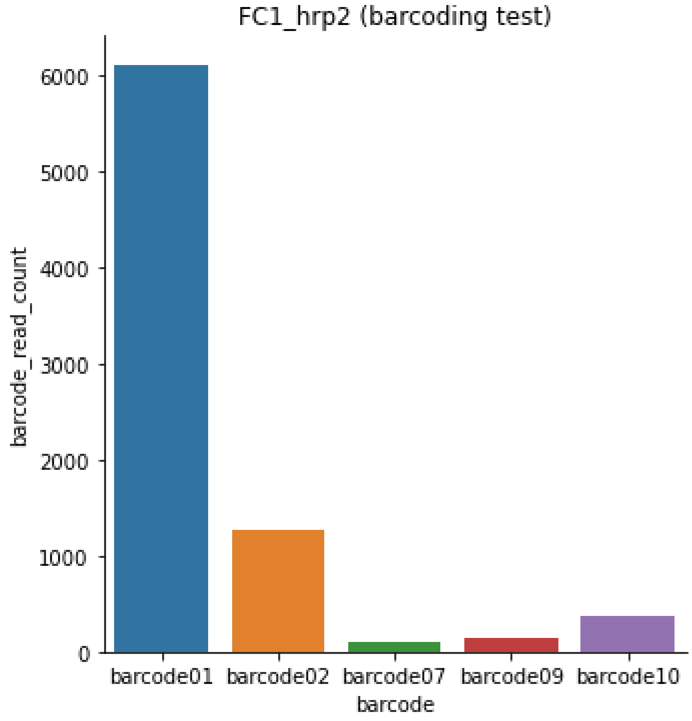

Supplementary Figure 3. Comparison of barcode distribution between *k13* and *pfhrp2* experiments. Sequencing runs "FC3_k13" and "FC1_hrp2" were performed on normalized replicates of control strain 7G8.

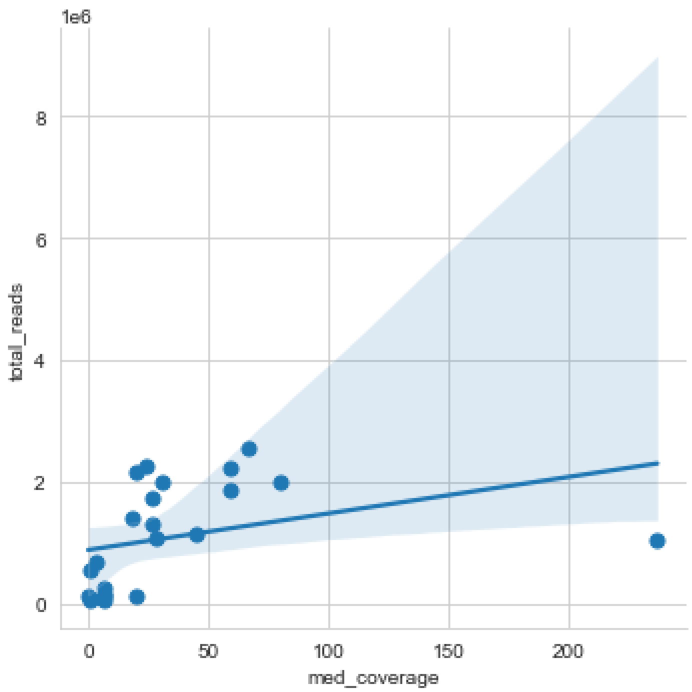

Supplementary Figure 4. There is a moderate relationship between the median coverage on *pfhrp2* from Dd2 negative controls (one per field sample evaluation sequencing run) and overall yield of passing reads in the sequencing runs. There is one outlier from run "FC7_valdom," where the median coverage of the Dd2 negative control reached 237x. This was still the lowest median coverage for all samples in the sequencing run.

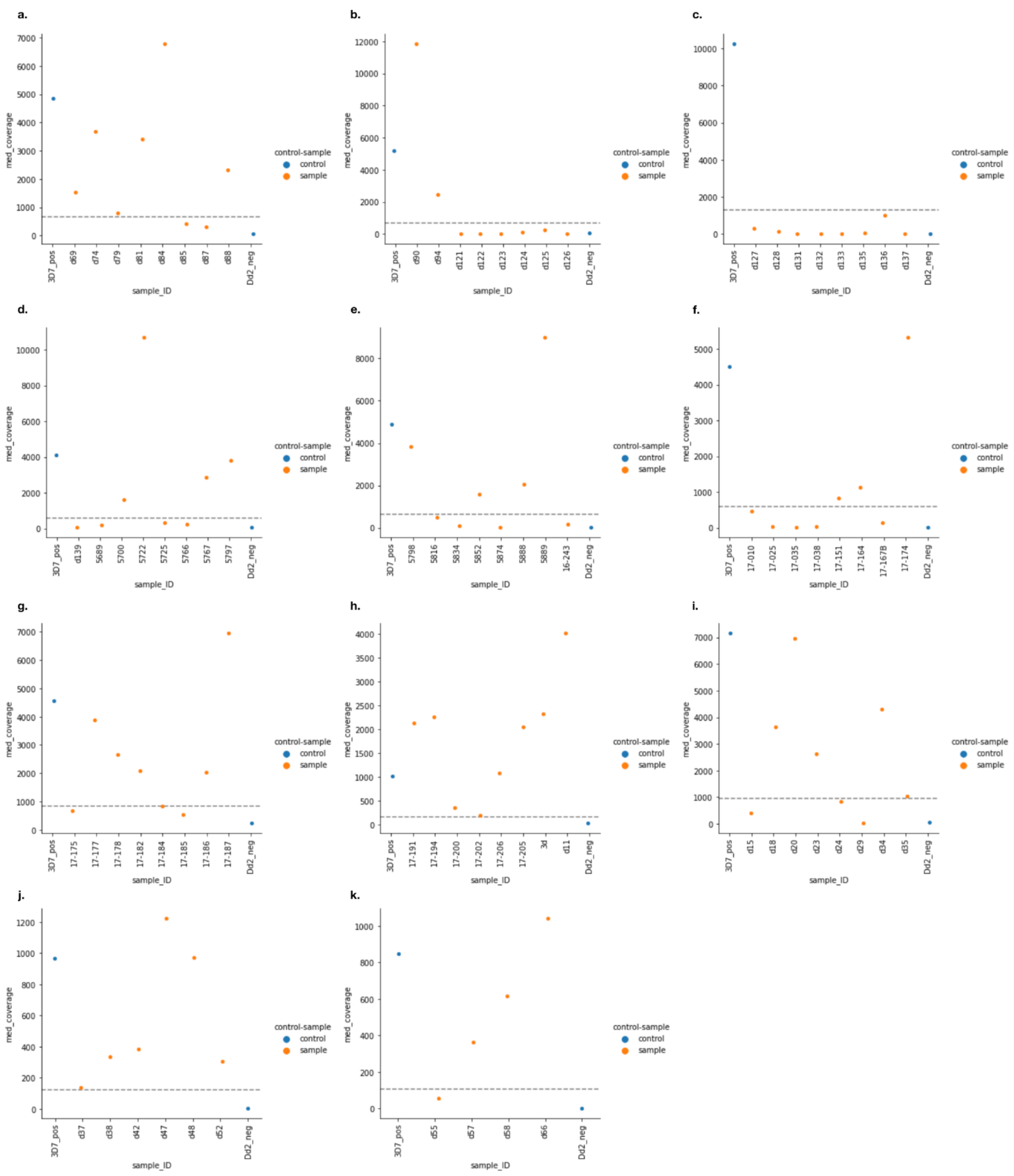
a.
med_coverage
0 1000 2000 3000 4000 5000 6000 7000
3D7_pos d69 d74 d79 d81 d84 d85 d87 d88 Dd2_neg
sample_ID
control-sample
control
sample
b.
med_coverage
0 2000 4000 6000 8000 10000 12000
3D7_pos d90 d94 d121 d122 d123 d124 d125 d126 Dd2_neg
sample_ID
control-sample
control
sample
c.
med_coverage
0 2000 4000 6000 8000 10000
3D7_pos d127 d128 d131 d132 d133 d135 d136 d137 Dd2_neg
sample_ID
control-sample
control
sample
d.
med_coverage
0 2000 4000 6000 8000 10000
3D7_pos d139 5689 5700 5722 5725 5766 5767 5797 Dd2_neg
sample_ID
control-sample
control
sample
e.
med_coverage
0 2000 4000 6000 8000
3D7_pos 5798 5816 5834 5852 5874 5888 5889 16-243 Dd2_neg
sample_ID
control-sample
control
sample
f.
med_coverage
0 1000 2000 3000 4000 5000
3D7_pos 17-010 17-025 17-035 17-038 17-151 17-164 17-1678 17-174 Dd2_neg
sample_ID
control-sample
control
sample
g.
med_coverage
0 1000 2000 3000 4000 5000 6000 7000
3D7_pos 17-175 17-177 17-178 17-182 17-184 17-185 17-186 17-187 Dd2_neg
sample_ID
control-sample
control
sample
h.
med_coverage
0 500 1000 1500 2000 2500 3000 3500 4000
3D7_pos 17-191 17-194 17-200 17-202 17-206 17-205 3d d11 Dd2_neg
sample_ID
control-sample
control
sample
i.
med_coverage
0 1000 2000 3000 4000 5000 6000 7000
3D7_pos d15 d18 d20 d23 d24 d29 d34 d35 Dd2_neg
sample_ID
control-sample
control
sample
j.
med_coverage
0 200 400 600 800 1000 1200
3D7_pos d37 d38 d42 d47 d48 d52 Dd2_neg
sample_ID
control-sample
control
sample
k.
med_coverage
0 200 400 600 800 1000
3D7_pos d55 d57 d58 d66 Dd2_neg
sample_ID
control-sample
control
sample

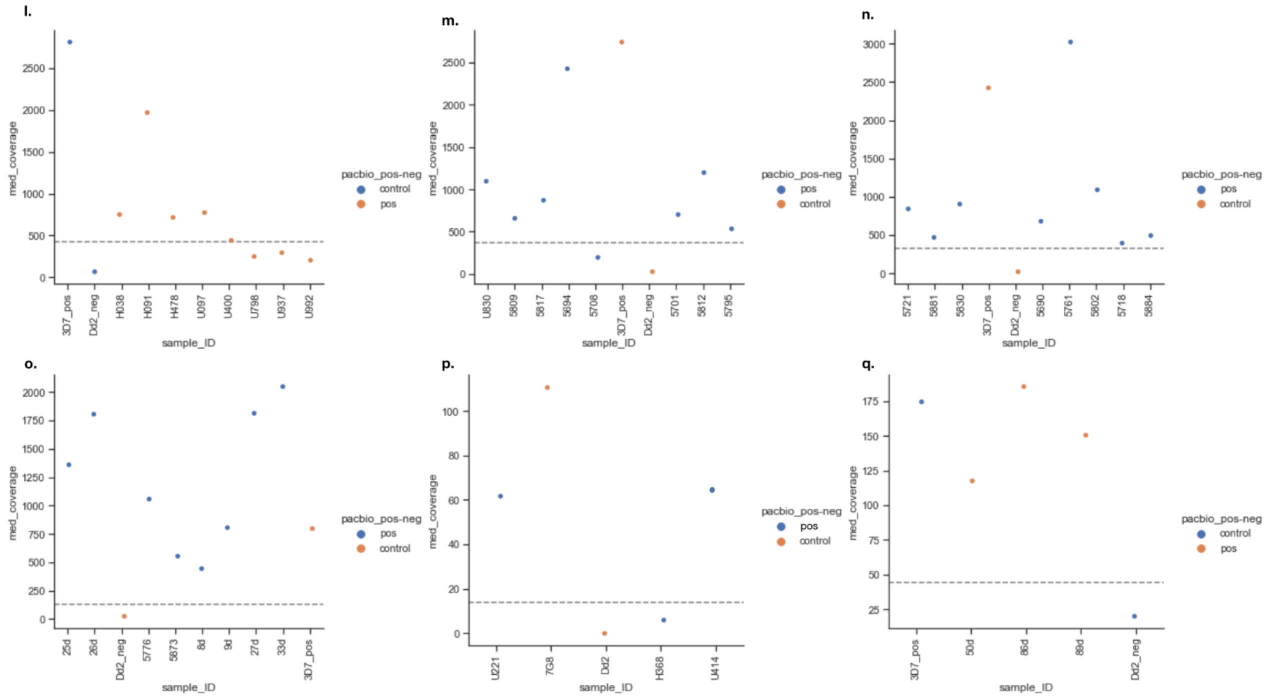

Supplementary Figure 5. Positivity threshold plots for the remaining field (a-k) and PacBio (l-q) samples not included in the TES sample set. For positivity thresholds for the TES sample set sequencing runs, see Figure 3.

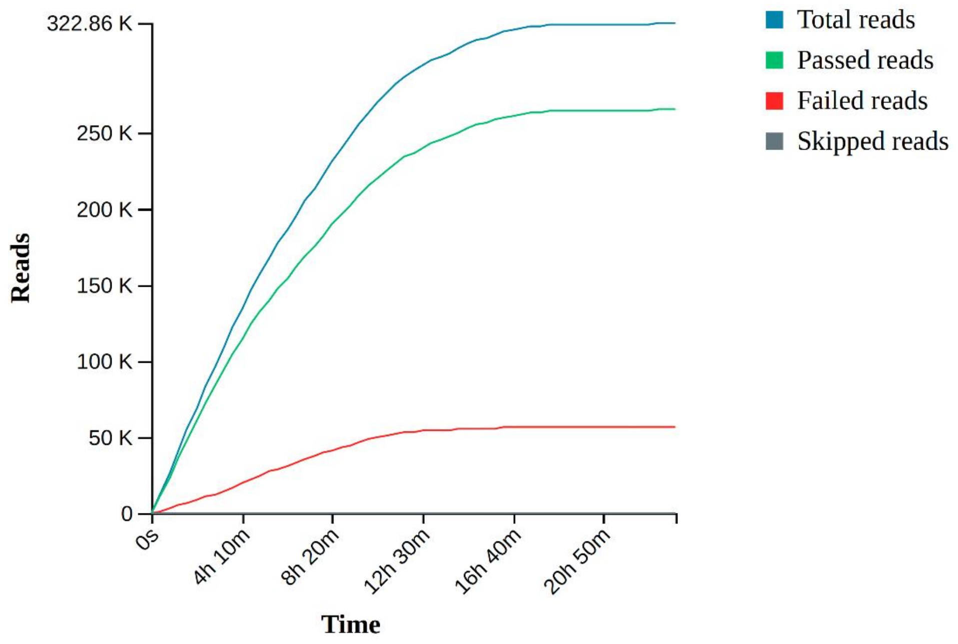

Supplementary Figure 6. Cumulative output reads from MinION sequencing report. When we ran a sequencing run (see FL1_TES in Supplementary Table 3) for 24 hours on a Flongle flow cell, we witnessed a plateau in data generation occur at approximately 12 hours.
